# Cellular and Molecular Mechanisms Underlie the Anti-Tumor Activities Exerted by *Walterinnesia aegyptia* Venom Combined with Silica Nanoparticles against Multiple Myeloma Cancer Cell Types

**DOI:** 10.1371/journal.pone.0051661

**Published:** 2012-12-10

**Authors:** Gamal Badr, Mohamed K. Al-Sadoon, Mostafa A. Abdel-Maksoud, Danny M. Rabah, Ahmed M. El-Toni

**Affiliations:** 1 Princess Johara Alibrahim Center for Cancer Research, Prostate Cancer Research Chair, College of Medicine, King Saud University, Riyadh, Saudi Arabia; 2 Immunology & Molecular Biology Laboratory, Zoology Department, Faculty of Science, Assiut University, Assiut, Egypt; 3 Zoology Department, College of Science, King Saud University, Riyadh, Saudi Arabia; 4 Department of Urology/Surgery, College of Medicine, King Saud University, Riyadh, Saudi Arabia; 5 King Abdullah Institute for Nanotechnology, King Saud University, Riyadh, Saudi Arabia; 6 Central Metallurgical Research and Development Institute, CMRDI, Helwan, Egypt; H.Lee Moffitt Cancer Center & Research Institute, United States of America

## Abstract

Multiple myeloma (MM) is a clonal disease of plasma cells that remains incurable despite the advent of several novel therapeutics. In this study, we aimed to delineate the impact of snake venom extracted from *Walterinnesia aegyptia* (WEV) alone or in combination with silica nanoparticles (WEV+NP) on primary MM cells isolated from patients diagnosed with MM as well as on two MM cell lines, U266 and RPMI 8226. The IC_50_ values of WEV and WEV+NP that significantly decreased MM cell viability without affecting the viability of normal peripheral mononuclear cells (PBMCs) were determined to be 25 ng/ml and 10 ng/ml, respectively. Although both WEV (25 ng/ml) and WEV+NP (10 ng/ml) decreased the CD54 surface expression without affecting the expression of CXCR4 (CXCL12 receptor) on MM cells, they significantly reduced the ability of CXC chemokine ligand 12 (CXCL12) to induce actin cytoskeleton rearrangement and the subsequent reduction in chemotaxis. It has been established that the binding of CXCL12 to its receptor CXCR4 activates multiple intracellular signal transduction pathways that regulate MM cell chemotaxis, adhesion, and proliferation. We found that WEV and WEV+NP clearly decreased the CXCL12/CXCR4-mediated activation of AKT, ERK, NFκB and Rho-A using western blot analysis; abrogated the CXCL12-mediated proliferation of MM cells using the CFSE assay; and induced apoptosis in MM cell as determined by PI/annexin V double staining followed by flow cytometry analysis. Monitoring the expression of B-cell CCL/Lymphoma 2 (Bcl-2) family members and their role in apoptosis induction after treatment with WEV or WEV+NP revealed that the combination of WEV with NP robustly decreased the expression of the anti-apoptotic effectors Bcl-2, Bcl_XL_ and Mcl-1; conversely increased the expression of the pro-apoptotic effectors Bak, Bax and Bim; and altered the mitochondrial membrane potential in MM cells. Taken together, our data reveal the biological effects of WEV and WEV+NP and the underlying mechanisms against myeloma cancer cells.

## Introduction

Hematologic malignancies are one of the most prevalent types of human cancers worldwide and cause high mortality rates. As the second most prevalent hematological cancer [1], multiple myeloma (MM) is a malignancy of plasma cells that afflicts approximately 20,000 and kills approximately 10,000 people in the United States annually [2]. Chemokines are a large family of low molecular weight (8–10 kDa) cytokine-like proteins that exhibit chemoattractant properties towards G-protein coupled seven-transmembrane receptors in leukocytes [3]. Several studies have revealed the important role of chemokines and their receptors in the pathogenesis of MM cells [4]. Chemokine receptors were demonstrated to be expressed on cancer cells and to act during all stages of tumor progression, including neoplastic transformation, chemotaxis and invasion, angiogenesis, clonal expansion and growth [5]. MM cells express variable levels of chemokine receptors [6]. Of the numerous expressed chemokine receptors, CXCR4 is the most highly expressed in MM and many other cancer cells [7]. The CXCR4 ligand, CXCL12, is strongly expressed in lung, liver, bone marrow and lymph nodes, which are all common metastatic destinations for many types of cancer. Moreover, the upregulation of CXCR4 has frequently been observed in various cancers, including colon carcinoma, lymphoma, breast cancer, glioblastoma, leukemia, prostate cancer, MM and pancreatic cancer [6]. Additionally, several studies have shown that CXCR4 is also the most abundant and functional of the chemokine receptors expressed by MM cells, and therefore, may play a major role in disease pathogenesis. Recent data suggest the involvement of CXCL12/CXCR4 in the maintenance and survival of MM cells in both in vivo and in vitro models [8]. Nevertheless, following the stimulation of CXCR4 with CXCL12 in MM cells, the activation of downstream signaling pathways remains obscure and the understanding of such signaling pathways represents an important molecular target for MM treatment [9]. Nuclear factor-κB (NF-κB) and AKT are involved in two major cell survival pathways that are often constitutively activated in cancer cells and contribute substantially to the chemoresistance of cancer cells [10]. However, the inhibition of ERK phosphorylation (another important cell survival pathway) contributes to dihydroartemisinin-induced apoptosis in liver cancer [11]. Our recent data demonstrated that thymoquinone (natural plant extract) induces MM cell growth arrest by abrogating CXCL12-mediated signaling and chemotaxis, as well as by increasing CD95 expression levels and the susceptibility of MM cells to Fas-mediated apoptosis [12]. Several reports have elucidated that failure to undergo apoptosis has been implicated in tumor development and resistance to cancer therapy [13]. Therefore, the induction of apoptosis in MM cells may lead to their regression and improved disease prognosis [14]. Thus, agents that are able to induce apoptosis may be useful chemotherapeutic agents against MM. In most tumor cells, apoptosis occurs via two different signaling pathways: the extrinsic and intrinsic apoptosis pathways. The intrinsic pathway is related to changes in the mitochondrial membrane potential (ΔΨm) [15], and therefore, mitochondrial membrane potential measurements could be used to discriminate between cells that have apoptosed and surviving cells. However, the Bcl-2 family of proteins consists of prominent regulators of apoptosis signaling that are often misappropriated in many cancers, including lung carcinoma, lymphoma, breast carcinoma and MM [16]. Members of this protein family can be divided into death antagonists, such as Bcl-2, and death agonists, such as Bak and Bax [17]. Of the Bcl-2 family, Bcl-2 is a prototypic anti-apoptotic protein that is frequently overexpressed in several types of human cancers [18]. Bcl-2 overexpression has been implicated in cancer chemoresistance, while high levels of pro-apoptotic proteins, such as Bax, promote apoptosis and sensitize tumor cells to various anti-cancer therapies.

Although the landscape of MM treatment is rapidly changing, this disease is largely incurable [19]. Several chemotherapeutic agents (e.g., vincristine, dexamethasone and melphalan) are currently used to treat MM. However, these drugs have the disadvantage of increasing the risk of developing secondary hematologic malignancies, such as therapy-related myelodysplastic syndromes [20]. Therefore, there is a crucial need to further identify biological factors and mechanisms that are responsible for MM cell survival, tumorigenesis and drug resistance [12].

Natural compounds have been used as adjuvants in combination with chemotherapy to reduce the side effects and increase the efficiency of cancer treatments [21]. In recent years, numerous natural products have been evaluated for their use in cancer treatment [22], [23]. Snake venom is a complex mixture of many substances with a wide spectrum of biological activities, including toxins, enzymes, growth factors, activators and inhibitors. Natural toxins, especially sub-lethal doses of snake venom, have the potential to reduce the size of solid tumors and block angiogenesis [24]. Our recent studies have demonstrated the anti-tumor potential of snake venom from *Walterinnesia aegyptia* (WEV) on the human breast carcinoma cell line MDA-MB-231, as well as its effect on normal mouse peripheral blood mononuclear cells (PBMCs) [25], [26]. Additionally, other data have indicated that *Vipera lebetina turanica* snake venom in the nanogram concentration range inhibits hormone-refractory human prostate cancer cell growth, and the effect is related to the NFκB signal-mediated induction of apoptosis [27]. Nanoparticles carrying chemical therapeutics have shown great promise in treating cancer patients. When loaded with anti-cancer agents, nanoparticles can successfully increase drug concentrations in cancer tissues and act at the cellular level to enhance anti-tumor efficacy. Nanoparticles can be endocytosed and/or phagocytosed by cells, resulting in the internalization of the encapsulated drug [28]. No data are available for the effects of snake venom in combination with nanoparticles on MM cancer cells. Therefore, in this study, we investigated the effects of *Walterinnesia aegyptia* venom (WEV), alone and in combination with silica nanoparticles (WEV+NP). We focused special attention on the cellular and molecular mechanisms underlying the anti-tumor activities exerted on the migration, invasion, proliferation and apoptosis of primary MM cells isolated from MM patients as well as 2 human MM cell lines (U266 and RPMI 8226).

## Materials and Methods

### Preparation of Walterinnesia Aegyptia Venom

*Walterinnesia aegyptia* snakes were collected from the central region of Saudi Arabia (No specific permits were required for the described field studies as well as no specific permissions were required for these locations/activities because the location is not privately-owned or protected in any way and the field studies did not involve endangered or protected species). The snakes were kept in a serpentarium in the Zoology Department of the College of Science at King Saud University. The snakes were warmed daily for nine hours using a 100-watt lamp, and water was always available. The snakes were fed purpose-bred mice every 10 to 14 days. All animal procedures were in accordance with the standards set forth in the guidelines for the care and use of experimental animals by the Committee for Purpose of Supervision of Experiments on Animals (CPCSEA) and the National Institutes of Health (NIH) protocol. The study protocol was approved by the Animal Ethics Committee of the Zoology Department, College of Science, King Saud University. The venom was milked from adult snakes, lyophilized and reconstituted in 1X phosphate-buffered saline (PBS) prior to use.

### Combination of Snake Venom with Silica Nanoparticles

Silica nanoparticles and their combination with snake venom were prepared at the King Abdullah Institute for Nanotechnology at King Saud University. Double mesoporous core-silica nanosphere shells were formed around silica cores using an anionic surfactant in a process in which a solid silica core was turned to a mesoporous one. First, for the synthesis of solid silica cores, 0.875 ml of aqueous ammonia was added to a solution containing 18 ml of ethanol and 2.6 ml of deionized water, followed by the addition of 1.5 ml of tetraethyl orthosilicate (TEOS) to the solution with vigorous stirring. The resulting mixture was heated at 30°C for 60 min, and the silica precipitate was then collected by centrifugation and washed three times with water. The molar composition of the suspension was as follows: TEOS: EtOH: NH_3_: H_2_O = 1∶46.9∶3.2∶20.5.

Secondly, for the synthesis of mesoporous core-shell nanospheres using an anionic surfactant, silica SiO_2_ particles were dispersed in 15 ml of H_2_O by ultrasonication for 10 min. To suppress silica core agglomeration, 1 g/L of polyvinylpyrrolidone was added with continuous stirring for 60 min. Thereafter, 0.1 ml, 0.2933 g (1 mmol) and 1.5 ml of 3-aminopropyltrimethoxysilane (APMS), N-lauroylsarcosine sodium (Sar-Na) and TEOS were added, respectively, to the reaction mixture with subsequent stirring at 50°C for 2 h. The final solid was recovered by centrifugation, washed with deionized water and dried in an oven at 60°C for 12 h Template removal was achieved by heat-treatment in an air stream at 550°C for 6 hours. The resulting molar ratio was TEOS: H_2_O: APMS: Sar-Na: HCl: PVP = 1∶331.6∶0.08∶0.14∶0.06∶5×10^−3^. Then, a total of 25 mg of mesoporous silica nanoparticles was added to a solution of 50 mg/ml venom in water. The suspension was stirred for 2 hours; the evaporation of water was prevented. Mesoporous silica nanoparticles loaded with venom were recovered using high-speed centrifugation and dried in a vacuum oven at 60°C. Transmission electron microscopy (TEM) analysis was performed using a JEOL JSM-2100F electron microscope (Japan) operated at 200 kV. Nitrogen sorption isotherms were measured at 77 K with a Quantachrome NOVA 4200 analyzer (USA). Prior to measuring, the samples were degassed in a vacuum at 200°C for at least 18 hours. The Brunauer-Emmett-Teller (BET) method was utilized to calculate the specific surface area (*S*BET) using adsorption data at a relative pressure range of 0.05 to 0.35. Using the Barrett-Joyner-Halenda (BJH) model, the pore volumes and pore size distributions were derived from the adsorption branches of isotherms, and the total pore volumes (*V*t) were estimated from the adsorbed amount at a relative pressure *P*/*P*0 of 0.992.

With respect to the effect of reaction time on the interaction between the silica nanoparticles and snake venom, variation in synthesis reaction time for solid silica cores resulted in the formation of double mesoporous core-shell silica spheres, as shown in Figure S 1. At both 1 and 6 h reaction times, homogenous mesoporous silica shells were observed. However, at a 1 h reaction time, the silica cores appeared to be quite dense. Extending the reaction time up to 6 h resulted in clear and pronounceable radially oriented mesopores that were anchored within the core and extended from the core center up to the shell, as shown in Figure S 1B. The decay of core contrast with increasing reaction time from 1 to 6 h suggested the anchoring of more mesopores and the loss of core density, which was also indicated by the high pore volume. However, the shell thickness of approximately 50 nm did not change significantly by tuning the reaction time. Analysis of the particle size distribution demonstrated that both samples displayed particle sizes approximately 300 nm. However, the 6 h reaction sample possessed a narrower distribution than the 1 h sample, as shown in Figure S 2. From the N_2_ adsorption–desorption isotherm shown in Figure S 3, the isotherms at different surfactant reaction times exhibited the type IV isotherm characteristic of mesoporous materials with total pore volumes of 0.342 and 0.416 cc.g^−1^ at the 1 and 6 h reaction times, respectively. The observation of a triangular hysteresis loop between the adsorption and desorption branches could be attributed to the coexistence of hexagonal and lamellar phases. The BET surface areas were 304.08 and 440.73 m^2^/g at the 1 and 6 h reaction times, respectively. It is clear that the textural characteristics of the mesoporous core-shell structures were enhanced with increasing reaction time.

### Cells and Reagents

The human MM cell lines RPMI8226 and U266 were obtained from the American Type Culture Collection (ATCC) (Rockville, MD). These MM cells were routinely maintained in R-10 culture media (RPMI 1640 containing 10% fetal calf serum and 1% L-glutamate), and cultures were established at 5×10^5^ viable cells/ml. Maximum cell density was established at 1–2×10^6^ cell/ml. Cell cultures were free of *Mycoplasma*, as assessed using an enzyme-linked immunosorbent assay (ELISA) kit (Boehringer, Mannheim, Germany).

Bone marrow (BM) aspirate samples from 70 patients with MM were obtained from the South Egypt Cancer Institute and the Assiut University Hospital of Assiut University in Egypt. Bone marrow mononuclear cells were isolated by Ficoll-Hypaque density gradient centrifugation (Pharmacia LKB Biotechnology, Uppsala, Sweden) from heparinized BM aspirate drawn from MM patients. Plasma cells were further purified from BM mononuclear cells by positive selection with anti-CD138 antibody-coated immunomagnetic beads according to the manufacturer’s instructions (MACS, Miltenyi Biotech). MACS purification consistently resulted in >95% purity in the primary MM cell population (by monitoring the cell-surface expression of CD38 and CD45) and a viability of >90% (by the trypan blue exclusion method).

PBMCs from healthy donors were purified using a standard Ficoll-Paque gradient centrifugation method according to the manufacturer’s instructions (Amersham Pharmacia, Uppsala, Sweden). Briefly, heparinized blood was diluted 1∶1 in phosphate-buffered saline (PBS), carefully layered over the Ficoll-Paque gradient, and centrifuged for 20 min at 1.020×*g*. The cell interface layer was carefully harvested, and the cells were washed twice in PBS and resuspended in RPMI 1640.

The anti-proliferative effect of WEV, WEV+NP or NP alone on the U266 and RPMI 8226 cell lines and the isolated normal PBMCs was determined using the 3-(4,5-dimethylthiazol-2-yl)-2,5-diphenyltetrazolium bromide (MTT) uptake method. The cells were plated at 1×10^6^ cells/ml in 2 ml of culture medium in six-well Costar plates (Corning, Corning, NY). The cells were treated with different concentrations of WEV or WEV+NP for 1, 2, 6, 12, 24, 36 or 48 h, and cytotoxicity was expressed as the relative percentage of the OD values measured in the control untreated (0), NP-, WEV- and WEV+NP-treated cells. Morphological changes were observed after exposure to NP, WEV and WEV+NP using a phase-contrast inverse microscope (Olympus, Japan).

### Ethics Statement

For snake collection from the central region of Saudi Arabia no specific permits were required for the described field studies as well as no specific permissions were required for these locations/activities because the location is not privately-owned or protected in any way and the field studies did not involve endangered or protected species. Because snakes were fed purpose-bred mice every 10 to 14 days, All animal procedures were in accordance with the standards set forth in the guidelines for the care and use of experimental animals by the Committee for Purpose of Supervision of Experiments on Animals (CPCSEA) and the National Institutes of Health (NIH) protocol. The study protocol was approved by the Animal Ethics Committee of the Zoology Department, College of Science, King Saud University. BM aspirate samples were obtained from patients identified with MM at Assiut University Hospital. All aspects of this study were approved by Assiut University’s ethical committee, and all patients provided a written informed consent accordingly to the Declaration of Helsinki.

### Flow Cytometry

Cell surface antigen expression was determined by single-parameter fluorescence-activated cell sorter (FACS) analysis using the following monoclonal antibodies (mAbs): (i) PE-conjugated anti-CCR1, anti-CCR3, anti-CCR5 (clone 45531.111), anti-CCR7 (clone 150503), anti-CCR6 (clone 53103.111), anti-CXCR3, anti-CXCR4 (clone 44717.111) and anti-CXCR5, all purchased from R&D Systems; and (ii) PE-conjugated anti-CD38, anti-CD44 and anti-CD138 (IgG1), FITC-conjugated anti-CD62L, anti-CD49d, anti-VLA4, anti-α4β1, anti-α4β7 and anti-CD54, and FITC- and PE-conjugated mouse isotype-matched control mAbs, all purchased from BD Biosciences. A FACSCalibur flow cytometry instrument (BD-PharMingen) was used for data acquisition and analysis. After viable cell gating, 15,000 events per sample were analyzed. For each marker, the threshold of positivity was defined beyond the nonspecific binding observed in the presence of a relevant isotype control mAb.

### F-actin Polymerization Assay

MM cells were cultured for 12 hours in culture medium supplemented with or without NP, WEV or WEV+NP before the F-actin polymerization test. Intracellular F-actin polymerization was assessed as previously described [29]. Briefly, cells were harvested and resuspended (4 × 10^6^/ml) in HEPES-buffered RPMI 1640 at 37°C with or without CXCL12 (250 ng/ml). At the indicated times, cell suspensions (100 µl) were added to 400 µl of assay buffer containing 4×10^−7^ M FITC-labeled phalloidin, 0.5 mg/ml L-α-lysophosphatidylcholine (both from Sigma-Aldrich) and 4.5% formaldehyde in PBS. The fixed cells were analyzed using flow cytometry, and the mean fluorescence intensity (MFI) was determined for each sample. The percentage change in MFI was calculated for each sample at each time point using the following formula: (1-(MFI before the addition of CXCL12/MFI after the addition of CXCL12) × 100.

### In Vitro Chemotaxis Assays

The chemokine-dependent migration of MM cells was measured using an in vitro 2-chamber migration assay (using Transwell plates purchased from Costar, Cambridge, MA) followed by flow cytometry analysis. All chemotaxis assays were performed in pre-warmed migration buffer (RPMI 1640 containing 1% FCS). A total of 600 µl of migration buffer alone or supplemented with CXCL12 (at 250 ng/ml) (R&D Systems) was added to the lower chamber, and 10^5^ cells in migration buffer were added to the upper chamber. The plates were then incubated for 3 h at 37°C, and input cells and transmigrated cells were centrifuged, fixed in 300 µl of 1×PBS+1% formaldehyde and counted for 60 seconds by flow cytometry using a FACSCalibur flow cytometer (BD-PharMingen). The percentage of migration was calculated as the percentage of input cells that migrated to the lower chamber. To calculate the percentage of specific migration induced by chemokines, the percentage of cells migrating to medium alone was subtracted from the percentage of cells migrating to medium with chemokine.

### Western Blot Analysis

Untreated (0) or NP-, WEV-, and WEV+NP-treated myeloma cells (5×106 cells/ml in pre-warmed R-10 medium without FCS) were stimulated for 2 min at 37°C with or without 250 ng/ml CXCL12. Lysates were prepared as previously described [29]. Equal amounts (50 µg) of total cellular protein were resolved using SDS-polyacrylamide gel electrophoresis (SDS-PAGE) and analyzed by western blotting. Antibodies recognizing phospho-PKB/AKT (S473), phospho-ERK1/2 (T202/Y204), phospho-IκBα (S32/36), IκBα, phospho-PLCβ3 (S537) and PLCβ3 (all from New England Biolabs, Beverly, MA) and anti-Bcl-2, Bcl_XL_, Mcl-1, Bax, Bak, Bim and β-actin antibodies (all from Santa Cruz Biotechnology, Santa Cruz, CA) were used in combination with horseradish peroxidase-conjugated secondary antibodies. Protein bands were detected using enhanced chemiluminescence reagents (ECL, Supersignal Westpico chemiluminescent substrate, Perbio, Bezons, France), and the ECL signal was recorded on hyperfilm ECL. To quantify band intensities, films were scanned, saved as TIFF files and analyzed using NIH Image J software.

To assess Rho-A activation, cells (5×10^6^ per condition) were starved for 2 h in pre-warmed R-10 medium without FCS, incubated for 2 min at 37°C with or without 250 ng/ml CXCL12, and lysed as described above (using 200 µl of lysis buffer). After centrifugation, a 15 µl aliquot of the supernatant was kept as the total lysate sample. The remaining supernatant (185 µl) was incubated for 16 h at 4°C with GST-C21^38^ pre-coupled to glutathione–agarose beads (Sigma, France). Beads were washed using an excess of lysis buffer, and the active Rho-A (Rho-A_GTP_ pulled down from the lysate and bound to the fusion protein/beads) was eluted using Laemli sample buffer. Analysis by SDS-PAGE and western blotting using an anti-Rho-A mAb (Santa Cruz Biotechnology, Santa Cruz, CA) was subsequently performed on both the total cell lysates and the samples eluted in Laemli buffer to detect total Rho-A and Rho-A_GTP_.

### CFSE Proliferation Assay

MM cells were harvested, washed twice in PBS and stained with 0.63 µM carboxyfluorescein diacetate succinimidyl ester (CFSE) (Molecular Probes, Eugene, OR) for 8 min at room temperature. Residual CFSE was removed by washing three times in PBS, and CFSE-labeled cells were seeded in 6-well plates, stimulated or not with CXCL12, treated without (0) or with NP, WEV and WEV+NP and grown for 4 days in cell culture medium. The CFSE fluorescence intensity was measured by flow cytometry using a FACSCalibur flow cytometer (BD-PharMingen).

### Apoptosis Assays

After treatment with NP, WEV and WEV+NP, myeloma cells were collected. Cells were washed and incubated in PBS containing 30% human AB serum at 4°C for 30 min prior to staining with Annexin V-FITC and PI for 15 min at 25°C using a commercial kit according to the manufacturer’s instructions (ABCam, Canada). Cells were analyzed by a FACSCalibur flow cytometer (BD-PharMingen) within 1 hour of staining, and the percentage of cells undergoing apoptosis was determined.

### Mitochondrial Membrane Potential Measurements

Mitochondrial energization was determined by the retention of JC-1dye (Molecular Probes). Briefly, MM cells (5×10^5^) were loaded with JC-1dye (1 µg/ml) during the last 30 min of incubation at 37°C in a 5% CO_2_ incubator. Cells were washed twice in PBS. Cells were then washed with FACS buffer, resuspended and stored in 500 µl of 2% paraformaldehyde solution. Approximately 10^5^ cells were analyzed by flow cytometry using a FACSCalibur flow cytometer (BD-PharMingen). Fluorescence was monitored in a fluorometer using 570 nm excitation/595 nm emission for the J-aggregate of JC-1. Mitochondrial membrane potential (Δψ_m_) was calculated as the ratio of the fluorescence of the JC-aggregate (aqueous phase) and monomer (membrane-bound) forms of JC-1. Therefore the use of JC-1 staining kitallowed us to discriminate between the apoptotic cells (green color) and surviving cells (red color).

### Statistical Analysis

Data were first tested for normality (using the Anderson–Darling test) and for homogeneity of variance prior to any further statistical analysis. Data were normally distributed and are expressed as the mean ± standard error of the mean (SEM). Significant differences among groups were analyzed using a one- or two-way ANOVA followed by Bonferroni’s multiple comparison tests using PRISM statistical analysis software (GraphPad Software). Data were reanalyzed using a one- or two-way ANOVA followed by Tukey’s post-test using SPSS (Statistical Package for the Social Sciences) software, version 17. Differences were considered statistically significant at P<0.05. *P<0.05, WEV-treated vs. control; #P<0.05, WEV+NP-treated vs. control; +P<0.05, WEV+NP-treated vs. WEV-treated.

## Results

### WEV and WEV+NP Inhibit the Growth of MM Cells

**Figure 1:**
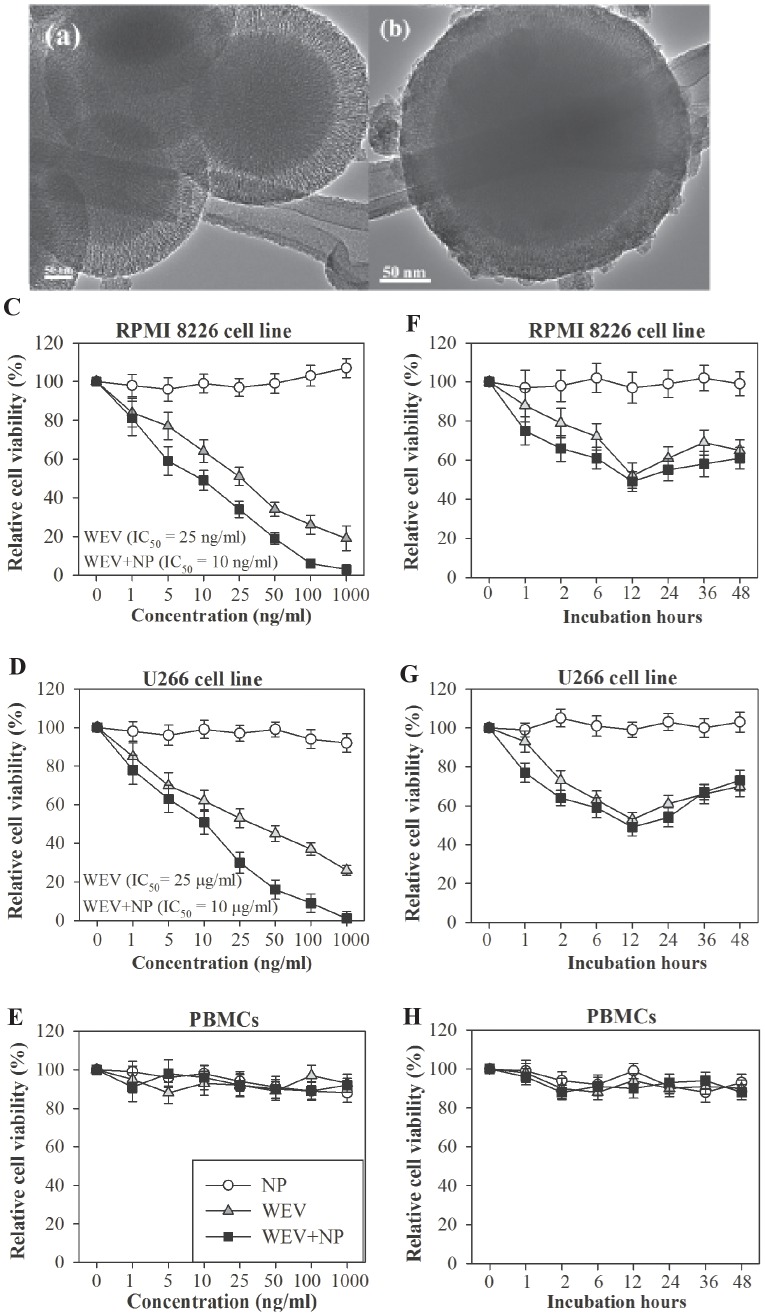
Time- and dose-dependent effect of WEV and WEV+NP treatment on cell viability. Electron microscope images of double mesoporous core-shell silica nanospheres (DMCSSs) before (**A**) and after (**B**) the venom loading. Cell viability was assessed using the MTT assay. RPMI 8226 cells (**C**), U266 cells (**D**) and normal PBMCs (**E**) were treated overnight with different concentrations (0, 1, 5, 10, 25, 50, 100 and 1000 ng/ml) of NP (open circles), WEV (gray triangles) or WEV+NP (closed black squares). RPMI 8226 cells (**F**), U266 cells (**G**) and normal PBMCs (**H**) were treated with 25 ng/ml of NP (open circles), 25 ng/ml of WEV (gray triangles) or 10 ng/ml of WEV+NP (closed black squares) for different incubation times (0, 1, 2, 6, 12, 24, 36 and 48 h). The data collected from independent experiments (n = 5) are shown, and the results are expressed as the mean percentage of viable cells ± SEM.

Electron microscope images of double mesoporous core-shell silica nanospheres (DMCSSs) before (**A**) and after (**B**) the venom loading are shown. These two images demonstrated an evidence for the loading of snake venom into mesopores of DMCSSs. Venom-free DMCSSs (**A**) demonstrated a clear mesopores or mesochannels. However, as shown in **Figure 1B** mesopores of DMCSSs have been clogged by the venom molecules and thus mesopores of venom-loaded DMCSS cannot be clearly seen due to the existence of venom inside their channels. In addition, the venom molecules can be also observed around the outer surface of DMCSSs. Zhao *et al.*, have reported that particle size for nanocarriers used in drug delivery systems should range between 50 and 300 nm [30]. The authors described that particle size above 300 nm, a significant proportion of particles will be trapped in the lungs and liver, while too small particle size will not be efficient for drug loading or drug delivery. Based on these criteria, DMCSSs-6h sample has been chosen for loading snake venom because it possessed superior textural properties, surface area of 440 m^2^/g and total pore volume of 0.416 cm^3^/g, thicker silica shell (40 nm) and acceptable particle size. Moreover, we recently characterized the optimal properties of silica nanoparticles model that we used to deliver natural products into MM cell line as well as into normal cells [31].

Using these nanoparticles model, We first investigated the ability of WEV and WEV+NP to induce growth arrest in MM cells. The effects of WEV and WEV+NP were examined on RPMI8226 (**Figure 1C**) and U266 (**Figure 1D**) MM cells and on normal human PBMCs (**Figure 1E**) at concentrations of 0, 1, 5, 10, 25, 50, 100 and 1000 µg/ml and incubation times of 0, 1, 2, 6, 12, 24, 36 and 48 h (**Figure 1F, G & H**). The resulting cytotoxic effects of WEV and WEV+NP were measured using the MTT uptake method. Data collected from several independent experiments (n = 5) demonstrated that WEV and WEV+NP significantly inhibited the growth of MM cells in a dose- and time-dependent manner. The IC_50_ values for WEV alone and WEV+NP were 25 µg/ml and 10 µg/ml, respectively. The effect was maximal at 12 h of incubation and decreased thereafter. The combination of WEV with NP (WEV+NP) significantly enhanced the inhibitory effect of WEV in MM cells. The maximal inhibitory effects of WEV and WEV+NP on cell viability were observed 12 h after treatment with 25 µg/ml of WEV alone or 10 µg/ml of WEV+NP. Nevertheless, treatment with WEV alone or WEV+NP did not produce significant cytotoxic effects on normal human PBMCs. Additionally, treatment with NP alone did not affect MM cell viability.

### WEV and WEV+NP Downregulate the Surface Expression of CD54 but not CXCR4 in MM Cells

**Figure 2:**
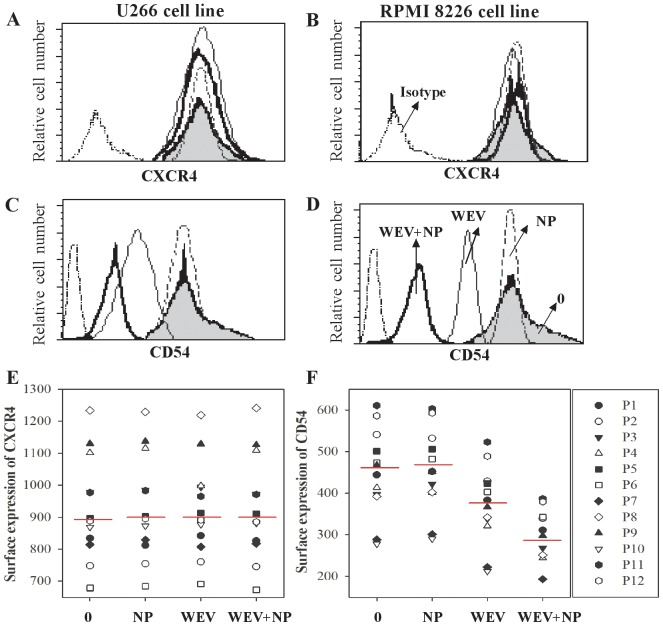
Effect of WEV and WEV+NP treatment on CXCR4 and CD54 expression in MM cells. MM cells were untreated (0) or treated with 25 ng/ml of NP, 25 ng/ml of WEV and 10 ng/ml of WEV+NP for 12 hours, and the surface expression of CXCR4 and CD54 was analyzed by flow cytometry using PE-conjugate mAbs. Histograms were gated based on viable cells, and one representative experiment is shown with the surface expression of CXCR4 (**A&B**) and CD54 (**C&D**) on U266 and RPMI 8226 cells, respectively. The results for the MFI of CXCR4 (**E**) and CD54 (**F**) expressed on primary MM cells isolated from patients with multiple myeloma that were untreated (0), or treated with NP, WEV and WEV+NP are shown (n = 12).

Chemokines, chemokine receptors and adhesion molecules play important roles in MM metastasis and progression. CD54, also known as intercellular adhesion molecule (ICAM)-1, is expressed in hematologic malignancies, including acute lymphoblastic leukemia (ALL), and plays a role in the homing of malignant plasma cells to the BM. Increased CD54 expression correlates with tumor cell growth in MM cells [32]. We therefore analyzed the surface expression of CXCR4 and CD54 following treatment with NP, WEV and WEV+NP on MM cells isolated from MM patients as well as on the RPMI8226 and U266 MM cell lines using flow cytometry. In one representative experiment, CXCR4 expression on RU266 (**Figure 2A**) and RPMI8226 cells (**Figure 2B**) was not altered by treatment with WEV, WEV+NP or NP. In contrast, CD54 expression was clearly downregulated in both U266 **Figure 2C**) and RPMI8226 cells (**Figure 2D**). Similarly, the treatment of MM cells from 12 MM patients with WEV alone or WEV+NP markedly downregulated the surface expression of CD54 (**Figure 2F**) but not CXCR4 (**Figure 2E**), compared to treatment with NP. Most importantly, WEV+NP (10 µg/ml) downregulated CD54 expression to a much greater extent than WEV alone (25 µg/ml). In contrast, treatment with NP had no effect on the expression levels of CXCR4 and CD54 on MM cells.

### WEV Combined with NP Decreases CXCL12-mediated Actin Polymerization and Cytoskeleton Rearrangement

**Figure 3:**
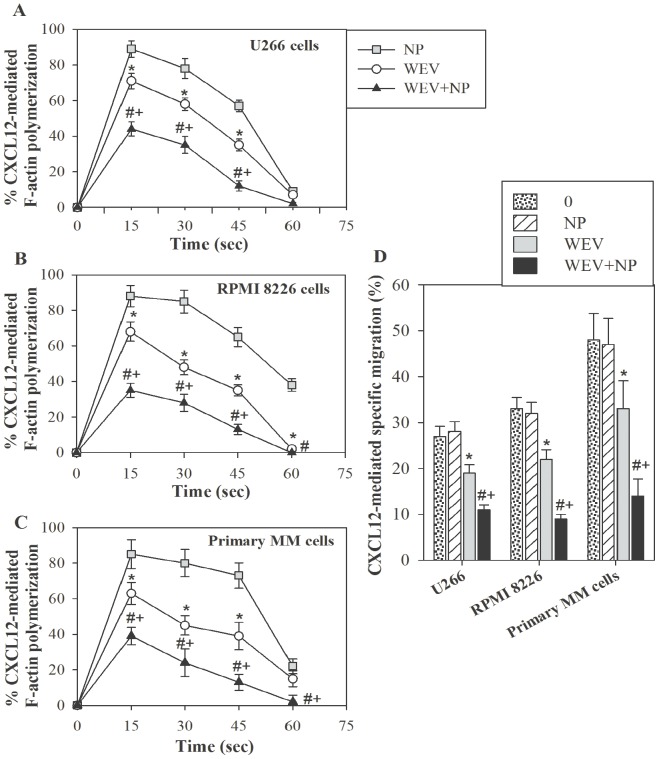
Effect of WEV and WEV+NP on cytoskeleton rearrangement. U266 (**A**), RPMI 8226 (**B**) and primary MM cells isolated from patients with multiple myeloma (**C**) were treated for 12 hours with 25 ng/ml of NP (gray squares), 25 ng/ml of WEV (open circles) or 10 ng/ml of WEV+NP (closed black triangles). Cells were then subjected to an F-actin polymerization assay following CXCL12 stimulation at the indicated times (15 second intervals), and the results were quantified using flow cytometry. The results are expressed as the mean percentage change in MFI (n = 12) ± SEM. (**D**) After treatment with NP, WEV and WEV+NP, the migratory response of U266, RPMI 8226 and primary MM cells to CXCL12 was determined using Transwell plates and flow cytometry analysis. The results are expressed as the mean percentage of specific migration ± SEM in response to CXCL12 (n = 12).

Cytoskeletal organization plays a central role in cell movement, migration, adhesion, proliferation, differentiation, vesicle trafficking and survival in both normal and malignant cells. Actin and microtubules provide a dynamic cellular framework that orchestrates and ultimately controls cellular activation and cancer metastasis. We therefore investigated whether WEV alone or in combination with NP affected actin polymerization after CXCL12 stimulation. After treatment of U266 (**Figure 3A**), RPMI 8226 (**Figure 3B**) and primary MM cells (**Figure 3C**) with NP, WEV (25 µg/ml) and WEV+NP (10 µg/ml) for 12 hours, the cells were stimulated every 15 sec with CXCL12 (250 ng/ml), and the degree of F-actin polymerization was determined using the F-actin assay and flow cytometry. The results are expressed as the mean percentage change in mean fluorescence intensity (MFI) ± SEM (n = 12). We found that both WEV alone and WEV+NP significantly decreased CXCL12-induced actin polymerization in MM cells compared to NP treatment. Most importantly, the treatment of MM cells with WEV+NP (10 µg/ml) decreased CXCL12-induced actin polymerization more than WEV alone (25 µg/ml). Because actin polymerization and cytoskeleton rearrangement are crucial processes in cell migration and chemotaxis, we then evaluated the effects of WEV and WEV+NP on CXCL12-mediated chemotaxis in MM cells, an essential phenomenon for the metastasis and survival of MM cells in the BM. After treatment with NP, WEV and WEV+NP, the migratory response of MM cells to CXCL12 was determined using Transwell plates and flow cytometry analysis. The results are expressed as the mean percentage of specific migration ± SEM in response to CXCL12 (n = 12). Data collected from 12 independent experiments performed on U266, RPMI8226 and primary MM cells revealed that WEV and WEV+NP treatments significantly reduced the CXCL12-mediated chemotaxis of MM cells (**Figure 3D**). Conversely, treatment of MM cells with NP had no effect on CXCL12-mediated actin polymerization and chemotaxis.

### Treatment with WEV and WEV+NP Decreased CXCL12/CXCR4-mediated Activation of AKT, ERK, NFκB and Rho-A

**Figure 4:**
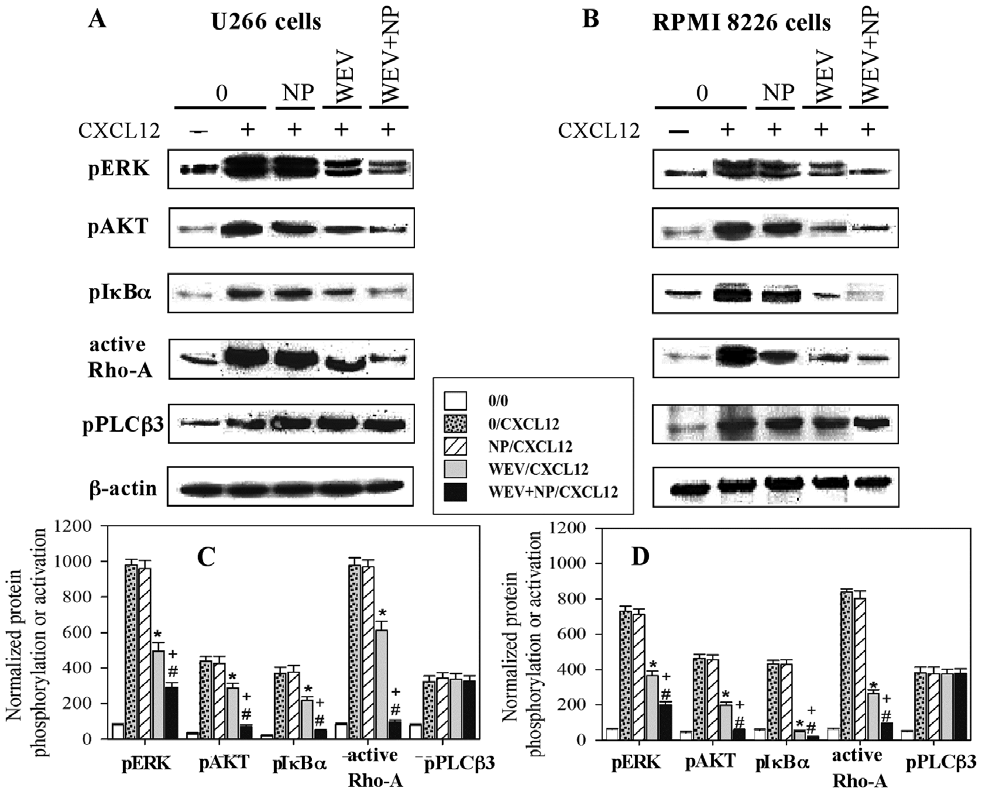
Effect of WEV and WEV+NP on CXCL12-mediated signaling. U266 (A) and RPMI 8226 (B) cells were untreated (0) or treated with 25 ng/ml of NP, 25 ng/ml of WEV or 10 ng/ml of WEV+NP for 12 hours. Cells were then treated for 2 min with medium or 250 ng/ml CXCL12 prior to being lysed. Proteins in the cell lysates were resolved on a 7% acrylamide gel. The phosphorylation levels of AKT (p-AKT), ERK1/2 (p-ERK1/2), IκBα (p- IκBα) and PLCβ3 (p-PLCβ3) and the activation level of Rho-A (Rho-A_GTP_) were corrected for the level of total β-actin on stripped blots. One representative blot for each downstream effector from 10 independent experiments is shown. Accumulated results are expressed as the mean values of normalized specific phosphorylation ± SEM from 10 separate experiments for U266 (**C**) and RPMI 8226 (**D**) cells. *P<0.05, WEV-treated vs. control; #P<0.05, WEV+NP-treated vs. control; +P<0.05, WEV+NP-treated vs. WEV-treated.

To better characterize the molecular mechanisms by which CXCL12 induces the chemotaxis of MM cells, we investigated the effect of CXCL12 on the activation of various downstream CXCR4 effectors. We recently demonstrated that CXCL12 mediated chemotaxis in MM cells by triggering the activation of PI3K/AKT/Rho-A, PLCβ3, NFκB and ERK1/2 signaling through its receptor CXCR4 [12]. Because treatment with WEV and WEV+NP significantly decreased CXCL12-mediated chemotaxis in MM cells, we therefore investigated the phosphorylation status of AKT, PLCβ3, IκBα and ERK1/2 and the activation status of Rho-A (an important adhesion protein in MM cells) following stimulation with CXCL12. The U266 (**Figure 4A**) and RPMI 8226 (**Figure 4B**) cell lines were not treated (0) or treated with NP, WEV (25 µg/ml) and WEV+NP (10 µg/ml) for 12 hours and then stimulated or not with CXCL12. The phosphorylation levels of AKT (p-AKT), ERK1/2 (p-ERK1/2), IκBα (p- IκBα) and PLCβ3 (p-PLCβ3) and the activation level of Rho-A (Rho-A_GTP_) were corrected for the total level of β-actin on stripped blots. The resulting protein bands for each downstream effector from 10 independent experiments are shown in one representative experiment in **Figure 4A** and **Figure 4B**. Although CXCL12 was able to upregulate the phosphorylation levels of ERK, AKT, IκBα and PLCβ3 and the activation level of Rho-A, treatment with WEV alone and WEV+NP clearly decreased the phosphorylation level of ERK, AKT, and IκBα and the activation of Rho-A but not the phosphorylation level of PLCβ3. The data from 10 independent experiments performed in U266 (**Figure 4C**) and RPMI8226 (**Figure 4D**) cells are expressed as the mean values of normalized specific phosphorylation and activation ± SEM. ^*^P<0.05, WEV-treated vs. control; ^#^P<0.05, WEV+NP-treated vs. control; ^+^P<0.05, WEV+NP-treated vs. WEV-treated. Our results reveal that treatment with WEV and WEV+NP significantly decreased (P<0.05) the CXCL12/CXCR4-mediated phosphorylation of AKT, ERK and NFκB and activation of Rho-A. It was observed that WEV+NP (10 µg/ml) decreased CXCL12 signaling in MM cells to a greater extent than WEV alone (25 µg/ml). Treatment with NP had no effect on the phosphorylation or activation of the effector transcription factors.

### WEV Combined with NP Abrogates the Proliferation of MM Cells

**Figure 5:**
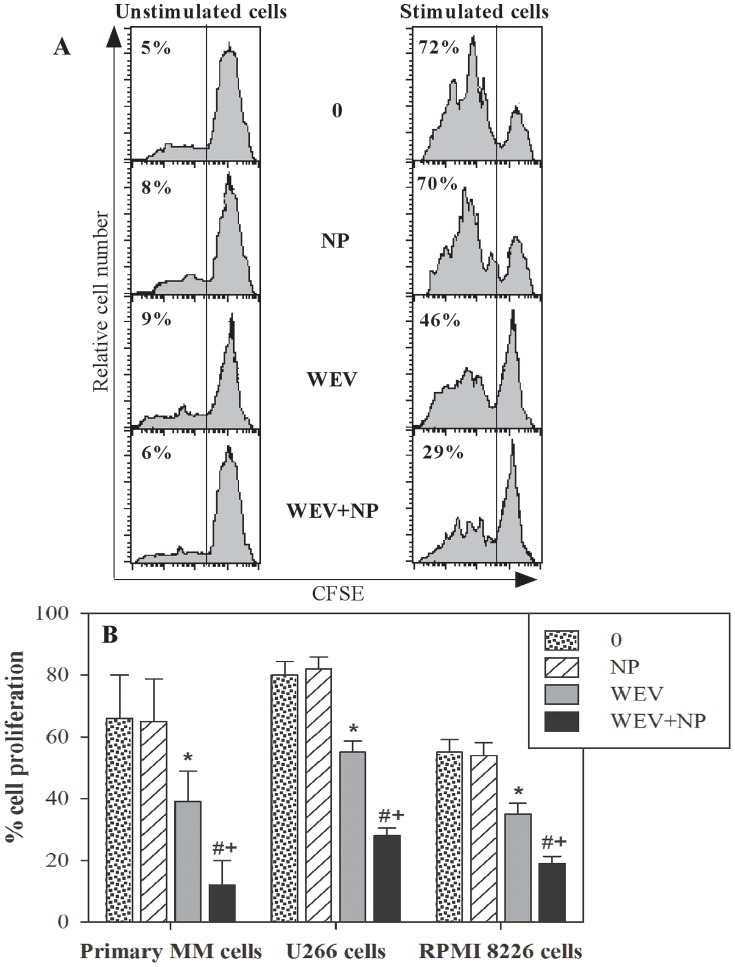
Effect of WEV and WEV+NP on MM cell proliferation. The ability of MM cancer cells to proliferate spontaneously or in response to CXCL12 stimulation was evaluated after treatment with NP, WEV and WEV+NP using the CFSE assay and flow cytometry analysis. (**A**) As shown in one representative experiment, analysis of CFSE staining was performed on U266 cells after gating the viable cells, and the percentage of proliferating cells (CFSE-lo) is indicated for each panel. (**B**) Data from independent experiments (n = 12) are expressed as the mean percentage of proliferating cells ± SEM in response to CXCL12. *P<0.05, WEV-treated vs. control; #P<0.05, WEV+NP-treated vs. control; ^+^P<0.05, WEV+NP-treated vs. WEV-treated.

Proliferation is a crucial process in the maintenance and progression of cancer cells. Therefore, we monitored the effects of WEV alone or combined with NP on the CXCL12-mediated proliferation of MM cells using a CFSE dilution assay followed by flow cytometry analysis. In one representative experiment, which is shown in **Figure 5A**, we found that the percentage of proliferating cells was markedly increased from 5% in unstimulated U266 cells to 72% in CXCL12-stimulated U266 cells. When U266 cells were treated with NP alone prior to stimulation with CXCL12, the percentage of proliferating cells was 70%, which is similar to the results obtained in untreated cells. However, treatment with WEV alone or combined with NP (WEV+NP) markedly decreased the percentage of proliferating cells to 46% and 29%, respectively. Data collected from 12 independent experiments revealed that treatment with WEV significantly reduced (P<0.05) the proliferative capacity of MM cells in response to CXCL12 (**Figure 5B**). Moreover, although NP had no effect, the inhibitory effect of WEV+NP on the CXCL12-induced proliferation of MM cells was stronger than that of WEV alone.

### WEV+NP Induces Apoptosis in MM Cells

**Figure 6:**
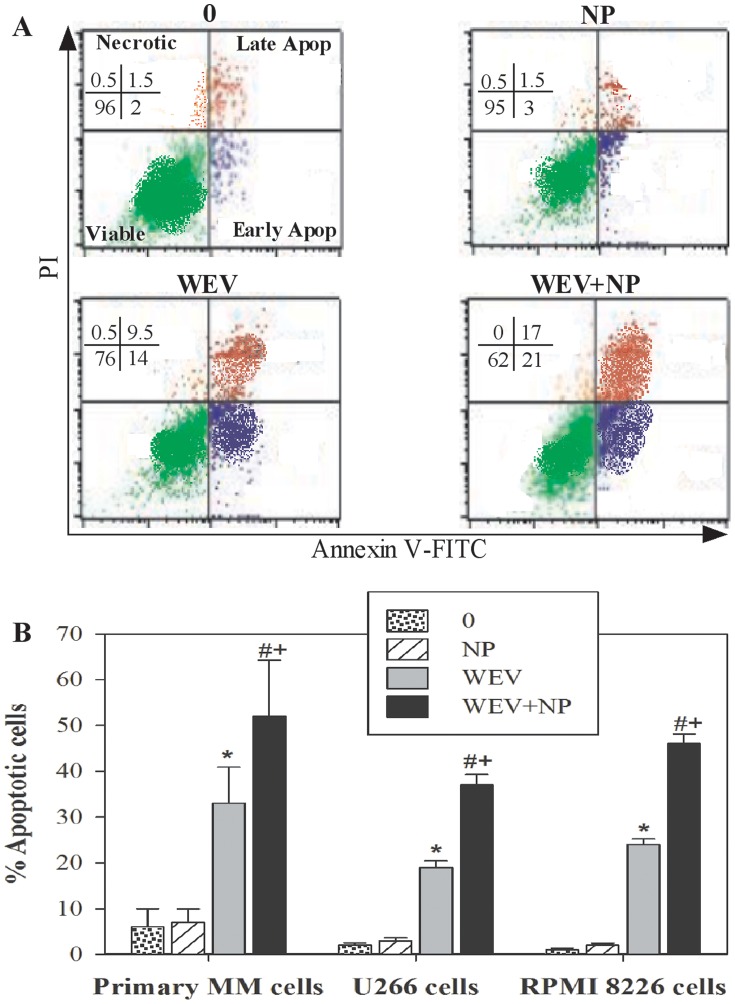
WEV alone or combined with NP induced apoptosis in MM cancer cells. The potential of WEV and WEV+NP to induce the apoptosis or necrosis of MM cancer cells was determined by flow cytometry based on their PI/Annexin V staining patterns. (**A**) One representative data set from 12 independent experiments is shown. (**B**) Accumulated data from 12 experiments are expressed as the mean percentage of apoptotic cells ± SEM for untreated cancer cells (0) (dotted bars), NP-treated cells (hatched bars), WEV-treated cells (closed gray bars) and WEV+NP-treated cells (closed black bars). ^*^P<0.05, WEV-treated vs. NP; ^#^P<0.05, WEV+NP-treated vs. NP; ^+^P<0.05, WEV+NP-treated vs. WEV-treated cells.

The inhibition of cancer cell proliferation, the cessation of cell cycle progression and the induction of apoptosis have all been targeted in chemotherapeutic strategies for the treatment of MM. We evaluated the impact of WEV and WEV+NP on apoptosis induction in MM cells using Annexin V/propidium iodide (PI) staining methods and flow cytometry analysis. As shown in **Figure 6A**, data from one representative experiment are presented in a dot plot demonstrating the discrimination between apoptotic, necrotic and viable cells. We found that the percentage of apoptotic cells was 3.5% and 4.5% in the untreated (0) and NP-treated U266 cells, respectively. Treatment with WEV and WEV+NP markedly increased the percentage of apoptotic cells to 123.5% and 38%, respectively. An increase in the induction of apoptosis after treatment with WEV and WEV+NP was inversely correlated with a decrease in the percentage of viable cells to 76% and 62% in WEV- and WEV+NP-treated U266 cells compared to 96% and 95% in untreated and NP-treated U266 cells, respectively. Data were pooled from different experiments (n = 12) performed on U266, RPMI8226 and primary MM cells isolated from 12 patients. The data indicate that treatment with WEV alone and WEV+NP significantly potentiated (P<0.05) apoptosis in MM cells (**Figure 6B**). Although NP had no significant effect on apoptosis induction in MM cells, the combination of NP with WEV significantly increased (P<0.05) apoptosis induction compared to WEV alone.

### WEV and WEV+NP Modulate the Expression of Bcl-2 Family Members and Alter the Mitochondrial Membrane Potential of MM Cells

**Figure 7:**
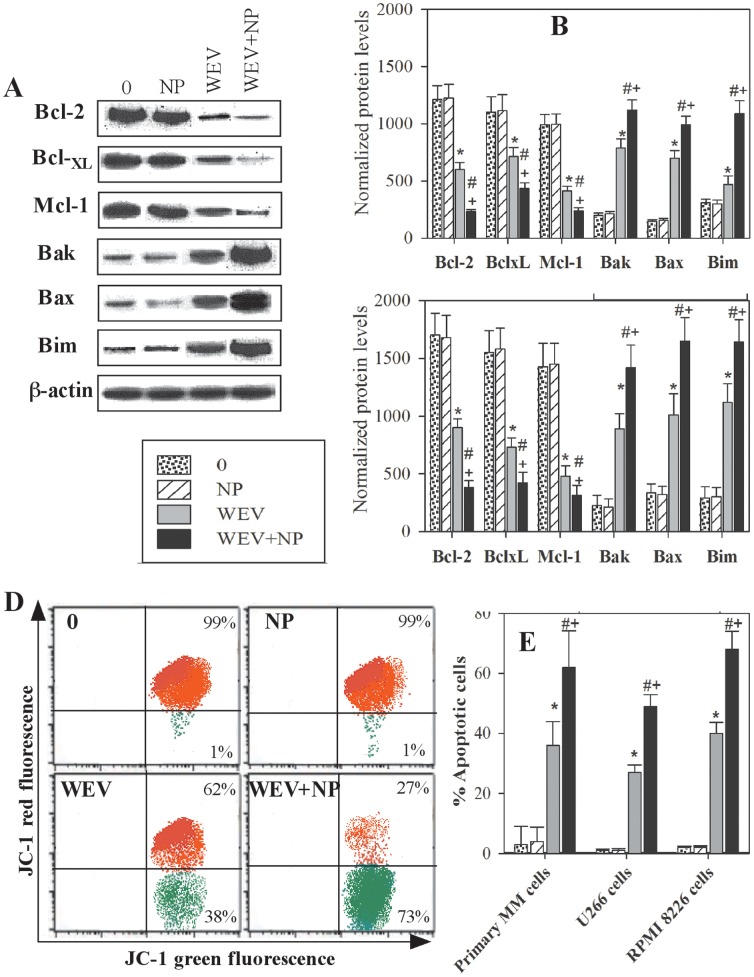
Effect of WEV and WEV+NP on the expression of Bcl-2 family members and mitochondrial membrane potential. The expression of Bcl-2 family members (anti-apoptotic proteins: Bcl-2, Bcl_XL_ and Mcl-1; and pro-apoptotic proteins: Bak, Bax and Bim) was monitored in the U266 and RPMI 8226 cell lines using western blot analysis. (**A**) Protein bands from one representative experiment performed using the U266 MM cell line are shown for cells that were untreated (0) or treated with NP, WEV or WEV+NP for 12 hours. (**B**) The expression of the anti-apoptotic and pro-apoptotic proteins was normalized to the total β-actin levels, and the results from 5 independent experiments performed using U266 cells are expressed as the mean ± SEM of the normalized values for the anti-apoptotic and pro-apoptotic proteins in untreated (dotted bars), NP-treated (hatched bars), WEV-treated (closed gray bars) and WEV+NP-treated (closed black bars) cells. (**C**) The expression of the anti-apoptotic and pro-apoptotic proteins was normalized to the total β-actin levels, and the results collected from 5 independent experiments performed on RPMI 8226 cells are expressed as the mean ± SEM of normalized values of the anti-apoptotic and pro-apoptotic proteins. Changes in the mitochondrial membrane potential were monitored using JC-1 and flow cytometry. (**D**) One representative experiment is shown, which discriminates between apoptotic (green dot plots) and surviving (red dot plots) U266 cells. (**E**) Accumulated data from 12 experiments are expressed as the mean percentage of apoptotic cells ± SEM for untreated cancer cells (0) (dotted bars), NP-treated cells (hatched bars), WEV-treated cells (closed gray bars) and WEV+NP-treated cells (closed black bars). ^*^P<0.05, WEV-treated vs. NP; ^#^P<0.05, WEV+NP-treated vs. NP; ^+^P<0.05, WEV+NP-treated vs. WEV-treated.

Bcl-2 family members play a central role in the regulation of apoptosis, proliferation and progression in cancer cells. Thus, the expression of Bcl-2 family members (the pro-apoptotic proteins Bak, Bax and Bim and the anti-apoptotic proteins Bcl-2, Bcl-_XL_ and Mcl-1) was monitored in the U266 and RPMI 8226 cell lines by western blot analysis. U266 cell lines were untreated (0) or treated for 12 h with NP, WEV and WEV+NP, and the resulting protein bands from one representative experiment are shown in **Figure 7A**. These protein bands clearly demonstrate that treatment with WEV alone and WEV+NP decreased the expression of anti-apoptotic Bcl-2 family proteins and inversely enhanced the expression of the pro-apoptotic proteins compared to untreated (0) and NP-treated cells. The expression of anti-apoptotic and pro-apoptotic proteins was normalized to the total β-actin protein level. The data from 5 independent experiments performed with the U266 (**Figure 7B**) and RPMI 8226 cell lines (**Figure 7C**) clearly illustrate that treatment with WEV alone (25 µg/ml) and WEV+NP (10 µg/ml) significantly abolished (P<0.05) the expression of the anti-apoptotic proteins Bcl-2, Bcl-_XL_ and Mcl-1. In contrast, a significant increase (P<0.05) in the expression of the pro-apoptotic proteins Bak, Bax and Bim was observed after treatment with WEV and WEV+NP. Furthermore, treatment with NP alone had no effect on the expression of the Bcl-2 family proteins.

Bcl-2 family proteins function upstream of irreversible cellular damage, and much of their activity occurs at the level of the mitochondrial membrane; thus, they play a pivotal role in determining if a cell lives or dies. We therefore monitored the changes in the mitochondrial membrane potential of MM cells following treatment with WEV and WEV+NP using JC-1 dye and flow cytometry analysis. **Figure 7D** shows one representative experiment with U266 cells after treatment with NP, WEV and WEV+NP; the dot plots show the discrimination between viable (red) and apoptotic (green) cells. The shift of cells from the upper panel (red dots) to the lower panel (green dots) indicates a decrease in the mitochondrial membrane potential and a subsequent increase in apoptosis induction in these cells. In this context, treatment with WEV and WEV+NP clearly increased the induction of apoptosis in MM cells. Data were pooled from experimental replicates (n = 12) performed on primary MM cells isolated from MM patients as well as on the U266 and RPMI 8226 cell lines (**Figure 7E**). The data clearly demonstrated that treatment with WEV alone (25 µg/ml) and WEV+NP (10 µg/ml) significantly diminished (P<0.05) the mitochondrial membrane potential. Although treatment with NP had no significant effect, the effect of WEV+NP treatment on the expression of Bcl-2 family proteins and the mitochondrial membrane potential was stronger than treatment with WEV alone.

## Discussion

Although snake venom has been previously reported to induce apoptosis in many cancer cell lines [27], [28], [33], there is presently no information on the effect of snake venom on human MM cells and its molecular mechanism of action. In this study, we aimed to delineate the impact of WEV alone or WEV+NP and clarify the underlying effector mechanisms in primary MM cells isolated from MM patients as well as in 2 MM cell lines, U266 and RPMI 8226. Pharmacologically targeting cell cycle arrest has been effectively used as a therapeutic strategy to restrict tumor growth in vitro and in vivo [34]. Here, we found that WEV alone or in combination with silica nanoparticles inhibited the growth of MM cells in a dose- and time-dependent manner. The effect peaked at 12 h and decreased thereafter indicating that WEV-mediated cytotoxicity effect on MM cells do not require *de novo* transcription. Moreover, the combination of WEV and NP (WEV+NP) enhanced the effect of WEV on these cancer cells. Furthermore, the IC_50_s of WEV and WEV+NP for the growth inhibition of MM cells were 25 µg/ml and 10 µg/ml, respectively. Similar results were observed in our recent study that demonstrated that the growth inhibition of breast cancer cells is related to apoptosis and cell cycle arrest [26].

Chemokines and their receptors play essential roles in the development, maintenance and proper functioning of the immune system. B cell-T cell interactions are modulated by chemokines. In B cell malignancies, these interactions may have tumor-promoting consequences [35]. In numerous studies, several chemokine receptors are viewed as promising therapeutic targets in the fight against cancer. Given its key role in mediating homing and retention of MM cells in the bone marrow, the CXCL12/CXCR4 axis was the natural target of such therapies. We therefore investigated, using flow cytometry analysis, the influence of WEV and WEV+NP on the CXCL12/CXCR4 axis in MM cells. Our results revealed that although both WEV (25 ng/ml) and WEV+NP (10 ng/ml) had no effects on the surface expression of CXCR4 (CXCL12 receptor), they significantly reduced the ability of CXCL12 to induce actin cytoskeleton rearrangement and the subsequent reduction in chemotaxis. In this context, recent studies showed the CXCL12 antagonist (AMD3100) or CXCR4 antagonist (T140) cause sensitization of MM cells to novel and standard chemotherapy agents by blocking the CXCL12/CXCR4 downstream signaling [36], [37]. Therefore, AMD3100 has been successfully used in phase I, II and III clinical studies to aid mobilization of CD34+ positive cells in patient undergoing harvesting for autologous bone marrow transplantation [38]. Our data demonstrated that WEV, either alone or combined with NP, altered the CXCL12-mediated signaling via CXCR4 rather than affecting the CXCR4 expression on MM cells. Similarly, we previously gp120 of HIV-1 inhibited CXCL12-mediated chemotaxis of human B cell [29]. This inhibition was mediated by blocking the intracellular signal transduction pathways downstream CXCR4, but did not impair the expression CXCR4.

CD54 is expressed on endothelial cells, epithelial cells, fibroblasts, leukocytes, and tumor cells.

High levels of CD54 expression have been found on some tumor cells, such as multiple myeloma [39]. Coleman et al, demonstrated that increased CD54 expression correlates with tumor cell growth in MM cells [32]. In this work the authors confirmed that myeloma cells with lower expressions of CD54 were more susceptible to apoptosis and the addition of CXCL12 to the in vitro culture significantly induced the up-regulation of CD54 expression in primary myeloma cells, promoting their survival. In addition, using UV3 which is a monoclonal antibody that recognizes human CD54, significantly prolonged the survival of xenograft mice model of MM with either early or advanced stages of disease. Therefore, it is possible that disrupting the CXCL12/CXCR4 axis blocks the CD54 on MM cells, increasing susceptibility to chemotherapy. Thus the effect of WEV and WEV+NP in decreasing the surface expression of CD54 could be of beneficial impact to blunt MM cell activation and enhancing susceptibility to chemotherapy. Similarly, it has been suggested that RGD-disintegrins isolated from snake venom are potent anti-metastatic agents that contribute to the inhibition of melanoma cell invasion [40]. Thus, by decreasing the chemotactic movement of MM cells, WEV or WEV+NP treatment is beneficial for fighting metastasis.

Uncontrolled proliferation has a major significance in cell turnover and tumorigenesis. CXCL12 attracts and induces proliferation of human MM cells and MM-derived cell lines, such as 5T33MM cells, to the endothelial border by activating CXCR4 [41]. Therefore, we monitored the effects of WEV alone or WEV combined with NP on the CXCL12-mediated proliferation of MM cells using the CFSE assay and detected abrogated CXCL12-mediated proliferation. The signaling pathways involved in CXCL12-mediated MM cell chemotaxis are poorly defined and require further clarification. CXCL12 binding to its receptor CXCR4 activates multiple intracellular signal transduction pathways that regulate MM cell chemotaxis, adhesion, proliferation and survival in BM. We previously demonstrated that direct stimulation of CXCR4 by CXCL12 activated two main signaling pathways in MM cells, PI3K and PLCβ3 [12]. PI3K in turn activates AKT, Rho-A, NFκB and ERK1/2, and the activation of these effectors were inhibited by using wortmannin (inhibitor of PI3K). Activation of PLCβ3 resulted in the rapid hydrolysis of phosphatidylinositol-4,5-bisphosphate, with production of inositol-1,4,5-trisphosphate and diacylglycerol, intracellular mediators that increase intracellular Ca2+ levels and activate protein kinase C activity. In the present study, activation of PI3K and PLCβ3 downstream CXCL12/CXCR4 axis has been confirmed. Therefore, using western blot analysis, we analyzed the effect of WEV and WEV+NP treatment on the signal transduction pathways downstream CXCR4. We found that WEV and WEV+NP clearly decreased the CXCL12/CXCR4-mediated activation of AKT, ERK, NFκB and Rho-A, but not of PLCβ3, suggesting that the signaling pathway of PI3K was more sensitive and was inhibited by treatment with WEV either alone or combined with NP. This phenomenon indicates that WEV partially, but not fully, inhibited the role of CXCL12/CXCR4 axis in MM cells. This hypothesis is clearly observed in the impact of WEV and WEV+NP on CXCL12-mediated actin polymerization and proliferation of MM cells. Moreover, WEV and WEV+NP induced cell cycle arrest by increasing the induction of apoptosis, as determined by Annexin V/PI double staining followed by flow cytometry analysis.

Next, we investigated the mechanism of cell cycle arrest exerted by this venom. Because Bcl-2 family members have been shown to be important regulators of malignant progression in many cancers [42], we used western blot analysis to detect the expression of Bcl-2 family proteins in MM cells isolated from patient samples and the U266 and RPMI 8226 cell lines after treatment with WEV and WEV+NP. Our data demonstrated that treatment with WEV alone or WEV combined with NP significantly diminished the expression of the anti-apoptotic Bcl-2 proteins Bcl-2, Bcl_XL_ and Mcl-1, whereas there was a significant increase in the expression of the pro-apoptotic Bcl-2 proteins Bak, Bax and Bim. Similar observations have been made by Park et al., who reported that snake venom toxin increases the expression of the pro-apoptotic protein Bax but downregulated the anti-apoptotic protein Bcl-2 [43]. Targeting Bcl-2 family proteins represents a promising strategy for the development of novel anti-cancer therapeutics. Therefore, the therapeutic potential of targeting the Bcl-2 signaling pathway is derived from the roles it plays in the promotion of cell growth and the inhibition of apoptosis. The interaction of the Bcl-2 family members at the mitochondrial outer membrane controls membrane permeability, and thus, the apoptotic program. Therefore, it was crucial to monitor the changes in the mitochondrial membrane potential after treatment with WEV and WEV+NP using JC-1 and flow cytometry analysis. We found that treatment with WEV and WEV+NP significantly decreased the mitochondrial membrane potential in MM cells. It has been reported that decreasing the mitochondrial membrane potential triggers multiple signaling cascades, including the activation of several caspases and the induction of apoptosis [44]. Other data have directly indicated a close relationship between the loss of mitochondrial membrane potential and the induction of apoptosis in MM cells [45]. Therefore, the ability of WEV and WEV+NP to affect Bcl-2 family protein expression, decrease mitochondrial membrane potential and subsequently induce apoptosis may also be mediated by a mechanism that sensitizes MM cells to chemotherapy but does not involve chemokines/chemokine receptor-mediated migration and invasion.

Generally, chemotherapeutic drugs attack on both normal and tumor cells non-specifically causing life threatening side effects, necessitating targeted drug delivery to tumors [46]. Indeed, the therapeutic molecule must generally: cross one or various biological membranes before diffusing through the plasma membrane to finally gain access to the appropriate organelle where the biological target is located. For those drugs whose target is located intracellularly, deviating from this ideal path may not only decrease the drug efficiency, but also entail side effects and toxicity. For these reasons, more than 30 years ago, the idea emerged to tailor carriers small enough to ferry the active substance to the target cell and its relevant subcellular compartment [47]. Various types of nanoparticles, such as liposomes, polymeric micelles, dendrimers, superparamagnetic iron oxide crystals, and colloidal gold, have been employed in targeted therapies for cancer [48], [49]. Nanocarriers offer unique possibilities to overcome cellular barriers in order to improve the delivery of various drug candidates [47]. To optimize the efficacy in delivery, often the tuning of physicochemical properties (i.e., particle size, shape, surface charge, lipophilicity, etc.) is necessary, in a manner specific to each type of nanoparticles. Recent studies showed an efficient tumor targeting by nanoparticles through the enhanced permeability and retention (EPR) effect [50]–[52]. Delivery of drug-loaded nanoparticles have achieved success in advanced thyroid cancer [53]. Here, while venom-free nanoparticles had no effect on MM cells, the combination of WEV with nanoparticles increased the efficiency of WEV to fight MM cells by two-fold when compared to WEV alone, although the IC_50_ of WEV+NP (10 µg/ml) was lower than that of WEV alone (25 µg/ml). Despite the large interest of using nanoparticles in biomedical applications, a clear understanding of their cellular uptake and transport is still lacking. They appear to translocate across cells via clathrin- and macropinocytosis-mediated endocytosis [54]. Experimental data agreed with that whereas PLL-g-PEG-DNA nanoparticles were internalized in an energy-dependent manner after 2 h and accumulated in the perinuclear region after more than 6 h [55]. In this study the authors demonstrated that the nanoparticles were found to be stable within the cytoplasm for at least 24 h and did not colocalize with the endosomal pathway. Furthermore, nanoparticle uptake was approximately 50% inhibited by genistein, an inhibitor of the caveolae-mediated pathway. However, clathrin-mediated endocytosis and macropinocytosis pathways were reduced by 17 and 24%, respectively, in the presence of the respective inhibitors. These findings suggest that PLL-g-PEG-DNA nanoparticles enter by several pathways and might therefore be an efficient and versatile tool to deliver therapeutic DNA [55]. The release of loaded drugs from nanoparticles may be controlled in response to changes in environmental condition such as temperature and pH. Biodistribution profiles and anticancer efficacy of nano-drugs in vivo would be different depending upon their size, surface charge, PEGylation and other biophysical properties [48]. Through the induction of T-cell immunity, nanoparticles may not only provide a therapeutic approaches for cancer but also provide a new prophylactic strategy for infectious disease [56], the reason why nanotechnology is expected to play a vital role in the rapidly developing field of nanomedicine, creating innovative solutions and therapies for currently untreatable diseases, and providing new tools for various biomedical applications, such as drug delivery and gene therapy [57].

## Supporting Information

Figure S1 **TEM images of calcined double mesoporous core-shell silica nanospheres prepared from silica cores formed at different reaction time of (a) 1 h and (b) 6 h.**
(TIF)

Figure S2 **Particle size distribution of calcined double mesoporous core-shell silica nanospheres prepared from silica cores formed at different reaction time of (a) 1 h and (b) 6 h.**
(TIF)

Figure S3 **N_2_ adsorption/desorption isotherms of calcined double mesoporous core-shell silica nanospheres prepared from silica cores formed at different reaction time of (a) 1 h and (b) 6 h.**
(TIF)

Figure S4 **N_2_ adsorption/desorption isotherms of calcined double mesoporous core-shell silica nanospheres prepared from silica cores formed at 6 h (a) before venom loading and (b) after venom loading.**
(TIF)

Figure S5 **TEM images of calcined double mesoporous core-shell silica nanospheres prepared from silica cores formed at 6 h (a) before venom loading and (b) after venom loading.**
(TIF)

## References

[1] Picot J, Cooper K, Bryant J, Clegg AJ (2011) The clinical effectiveness and cost-effectiveness of bortezomib and thalidomide in combination regimens with an alkylating agent and a corticosteroid for the first-line treatment of multiple myeloma: a systematic review and economic evaluation. Health Technol Assess 15: 1–204. 10.3310/hta15410 PMC4781217 22146234

[2] Tuchman SA, Chao NJ, Gasparetto CG (2012) Lenalidomide before and after Autologous Hematopoietic Stem Cell Transplantation in Multiple Myeloma. Adv Hematol 2012: 712613. 22690220 10.1155/2012/712613 PMC3368529

[3] Yoshie O, Imai T, Nomiyama H (2001) Chemokines in immunity. Adv Immunol 78: 57–110. 11432208 10.1016/s0065-2776(01)78002-9

[4] Aggarwal R, Ghobrial IM, Roodman GD (2006) Chemokines in multiple myeloma. Exp Hematol 34: 1289–1295. 16982321 10.1016/j.exphem.2006.06.017 PMC3134145

[5] Arya M, Patel HRH, Williamson M (2003) Chemokines key players in cancer. Curr Med Res Opin 19: 557–564. 14594528 10.1185/030079903125002216

[6] Moller C, Stromberg T, Juremalm M, Nilsson K, Nilsson G (2003) Expression and function of chemokine receptors in human multiple myeloma. Leukemia 17: 203–210. 12529679 10.1038/sj.leu.2402717

[7] Fulton AM (2009) The chemokine receptors CXCR4 and CXCR3 in cancer. Curr Oncol Rep 11: 125–131. 19216844 10.1007/s11912-009-0019-1

[8] Alsayed Y, Ngo H, Runnels J, Leleu X, Singha UK (2007) Mechanisms of regulation of CXCR4/SDF-1 (CXCL12)-dependent migration and homing in multiple myeloma. Blood 109: 2708–2717. 17119115 10.1182/blood-2006-07-035857 PMC1852222

[9] Rossi M, Di Martino MT, Morelli E, Leotta M, Rizzo A, et al (2012) Molecular targets for the treatment of Multiple myeloma. Curr Cancer Drug Targets 12: 757–767. 22671925 10.2174/156800912802429300

[10] Sun H, Zheng X, Wang Q, Yan J, Li D, et al (2012) Concurrent blockade of NFκB and AKT pathways potentiates cisplatin’s antitumor activity in vivo. Anticancer Drugs 23: 1039–1046. 22760211 10.1097/CAD.0b013e32835679b8

[11] Zhang CZ, Pan Y, Cao Y, Lai PB, Liu L, et al (2012) Histone deacetylase inhibitors facilitate dihydroartemisinin-induced apoptosis in liver cancer in vitro and in vivo. PLoS One 7: e39870. 22761917 10.1371/journal.pone.0039870 PMC3386188

[12] Badr G, Lefevre EA, Mohany M (2011) Thymoquinone inhibits the CXCL12-induced chemotaxis of multiple myeloma cells and increases their susceptibility to Fas-mediated apoptosis. PLoS One 6: e23741. 21912642 10.1371/journal.pone.0023741 PMC3164673

[13] Tiberio P, Cavadini E, Callari M, Daidone MG, Appierto V (2012) AF1q: A Novel Mediator of Basal and 4-HPR-Induced Apoptosis in Ovarian Cancer Cells. PLoS One 7: e39968. 22761939 10.1371/journal.pone.0039968 PMC3383705

[14] Bruckheimer EM, Kyprianou N (2000) Apoptosis in prostate carcinogenesis. A growth regulator and a therapeutic target. Cell Tissue Res 301: 153–162. 10928288 10.1007/s004410000196

[15] Zhang A, Wu Y, Lai HWL, Yew DT (2004) Apoptosis a brief review. Neuroembryology 3: 47–59.

[16] Kouri FM, Jensen SA, Stegh AH (2012) The role of Bcl-2 family proteins in therapy responses of malignant astrocytic gliomas: BCL-2L12 and beyond. Scientific World Journal 2012: 838916. 22431925 10.1100/2012/838916 PMC3289992

[17] Oltvai ZN, Milliman CL, Korsmeyer SJ (1993) Bcl-2 heterodimerizes in vivo with a conserved homolog, Bax, that accelerates programmed cell death. Cell 74: 609–619. 8358790 10.1016/0092-8674(93)90509-o

[18] Gao Q, Yang S, Kang MQ (2012) Influence of survivin and Bcl-2 expression on the biological behavior of non-small cell lung cancer. Mol Med Report 5: 1409–1414. 10.3892/mmr.2012.840 22446832

[19] Nishihori T, Alsina M (2011) Advances in the autologous and allogeneic transplantation strategies for multiple myeloma. Cancer Control 18: 258–267. 21976244 10.1177/107327481101800406

[20] Ishii Y, Hsiao HH, Sashida G, Ito Y, Miyazawa K (2006) Derivative (1;7)(q10;p10) in multiple myeloma. A sign of therapy-related hidden myelodysplastic syndrome. Cancer Genet Cytogenet 167: 131–137. 16737912 10.1016/j.cancergencyto.2006.01.002

[21] Ladas EJ, Jacobson JS, Kennedy DD, Teel K, Fleischauer A (2004) Antioxidants and cancer therapy: a systematic review. J Clin Oncol 22: 517–528. 14752075 10.1200/JCO.2004.03.086

[22] Heiferman MJ, Salabat MR, Ujiki MB, Strouch MJ, Cheon EC (2010) Sansalvamide induces pancreatic cancer growth arrest through changes in the cell cycle. Anticancer Res 30: 73–78. 20150619

[23] Lam M, Carmichael AR, Griffiths HR (2012) An Aqueous Extract of Fagonia cretica Induces DNA Damage, Cell Cycle Arrest and Apoptosis in Breast Cancer Cells via FOXO3a and p53 Expression. PLoS One 7: e40152. 22761954 10.1371/journal.pone.0040152 PMC3384610

[24] Francis S, Markland A (2001) Novel Snake venom disintegrin that inhibits human ovarian cancer dissemination and angiogenesis in an orthotopic nude mouse model. Haemostasis 31: 183–191. 11910184 10.1159/000048062

[25] Badr G, Al-Sadoon MK, El-Toni AM, Daghestani M (2012) Walterinnesia aegyptia venom combined with silica nanoparticles enhances the functioning of normal lymphocytes through PI3K/AKT, NFκB and ERK signaling. Lipids Health Dis 2012: 11–27. 10.1186/1476-511X-11-27 PMC3310743 22336518

[26] Al-Sadoon MK, Abdel-Maksoud MA, Rabah DM, Badr G (2012) Induction of Apoptosis and Growth Arrest in Human Breast Carcinoma Cells by a Snake (Walterinnesia aegyptia) Venom Combined With Silica Nanoparticles: Crosstalk Between Bcl2 and Caspase 3. Cell Physiol Biochem 30: 653–665. 22854437 10.1159/000341446

[27] Son DJ, Park MH, Chae SJ, Moon SO, Lee JW, et al (2007) Inhibitory effect of snake venom toxin from Vipera lebetina turanica on hormone-refractory human prostate cancer cell growth: induction of apoptosis through inactivation of nuclear factor kappa B. Mol Cancer Ther. 6: 675–83. 10.1158/1535-7163.MCT-06-0328 17308063

[28] Barratt G (2003) Colloidal drug carriers: achievements and perspectives. Cell Mol Life Sci 60: 21–37. 12613656 10.1007/s000180300002 PMC11138927

[29] Badr G, Borhis G, Treton D, Moog C, Garraud O, et al (2005) HIV type 1 glycoprotein 120 inhibits human B cell chemotaxis to CXC chemokine ligand (CXCL) 12, CC chemokine ligand (CCL) 20, and CCL21. J Immunol 175: 302–310. 15972662 10.4049/jimmunol.175.1.302

[30] Zhao W, Gu J, Zhang L, Chen H, Shi J (2005) Fabrication of uniform magnetic nanocomposite spheres with a magnetic core/mesoporous silica shell structure. J Am Chem Soc 127: 8916–8917. 15969545 10.1021/ja051113r

[31] El-Toni AM, Khan A, Ibrahim MA, Labis JP, badr G, et al (2012) Synthesis of double mesoporous core-shell silica spheres with tunable core porosity and their drug release and cancer cell apoptosis properties. J Colloid Interface Sci 378: 83–92. 22551476 10.1016/j.jcis.2012.04.006

[32] Coleman EJ, Brooks KJ, Smallshaw JE, Vitetta ES (2006) The Fc portion of UV3, an anti-CD54 monoclonal antibody, is critical for its antitumor activity in SCID mice with human multiple myeloma or lymphoma cell lines. J Immunother 29: 489–498. 16971805 10.1097/01.cji.0000210079.52554.c3

[33] Park MH, Jo MR, Won D, Song HS, Han SB, et al (2012) Snake venom toxin from Vipera lebetina turanica induces apoptosis in colon cancer cells via upregulation of ROS- and JNK-mediated death receptor expression. BMC Cancer 12(1): 228. 22681760 10.1186/1471-2407-12-228 PMC3584847

[34] Song JK, Jo MR, Park MH, Song HS, An BJ, et al (2012) Cell growth inhibition and induction of apoptosis by snake venom toxin in ovarian cancer cell via inactivation of nuclear factor κB and signal transducer and activator of transcription 3. Arch Pharm Res 35: 867–876. 22644854 10.1007/s12272-012-0512-1

[35] Jöhrer K, Hofbauer SW, Zelle-Rieser C, Greil R, Hartmann TN (2012) Chemokine-dependent B cell-T cell interactions in chronic lymphocytic leukemia and multiple myeloma - targets for therapeutic intervention? Expert Opin Biol Ther 12: 425–441. 22332909 10.1517/14712598.2012.664128

[36] Azab AK, Runnels JM, Pitsillides C, Moreau AS, Azab F, et al (2009) CXCR4 inhibitor AMD3100 disrupts the interaction of multiple myeloma cells with the bone marrow microenvironment and enhances their sensitivity to therapy. Blood 113: 4341–4351. 19139079 10.1182/blood-2008-10-186668 PMC2676090

[37] Zhang WB, Navenot JM, Haribabu B, Tamamura H, Hiramatu K, et al (2002) A point mutation that confers constitutive activity to CXCR4 reveals that T140 is an inverse agonist and that AMD3100 and ALX40–4C are weak partial agonists. J Biol Chem 277: 24515–24521. 11923301 10.1074/jbc.M200889200

[38] Pelus LM, Horowitz D, Cooper SC, King AG (2002) Peripheral blood stem cell mobilization. A role for CXC chemokines. Crit Rev Oncol Hematol 43: 257–275. 12270782 10.1016/s1040-8428(01)00202-5

[39] Yang L, Froio RM, Sciuto TE, Dvorak AM, Alon R, et al (2005) ICAM-1 regulates neutrophil adhesion and transcellular migration of TNF-alpha-activated vascular endothelium under flow. Blood 106: 584–592. 15811956 10.1182/blood-2004-12-4942 PMC1635241

[40] Oliva IB, Coelho RM, Barcellos GG, Saldanha-Gama R, Wermelinger LS, et al (2007) Effect of RGD disintegrins on melanoma cell growth and metastasis: involvement of the actin cytoskeleton, FAK and c-Fos. Toxicon 50: 1053–1063. 17854854 10.1016/j.toxicon.2007.07.016

[41] Menu E, Asosingh K, Indraccolo S, De Raeve H, Van Riet I (2006) The involvement of stromal derived factor 1alpha in homing and progression of multiple myeloma in the 5TMM model. Haematologica 91: 605–612. 16627256

[42] Song JH, Kraft AS (2012) Pim kinase inhibitors sensitize prostate cancer cells to apoptosis triggered by Bcl-2 family inhibitor ABT-737. Cancer Res 72: 294–303. 22080570 10.1158/0008-5472.CAN-11-3240 PMC3251634

[43] Park MH, Son DJ, Kwak DH, Song HS, Oh KW, et al (2009) Snake venom toxin inhibits cell growth through induction of apoptosis in neuroblastoma cells. Arch Pharm Res 32: 1545–1554. 20091267 10.1007/s12272-009-2106-0

[44] Thornberry NA, Lazebnik Y (1998) Caspases: enemies within. Science 281: 1312–1316. 9721091 10.1126/science.281.5381.1312

[45] Salido M, Gonzalez JL, Vilches J (2007) Loss of mitochondrial membrane potential is inhibited by bombesin in etoposide-induced apoptosis in PC-3 prostate carcinoma cells. Mol Cancer Ther 6: 1292–1299. 17431107 10.1158/1535-7163.MCT-06-0681

[46] Kolluru LP, Rizvi SA, D’Souza M, D’Souza MJ (2012) Formulation development of albumin based theragnostic nanoparticles as a potential delivery system for tumor targeting. J Drug Target. 2012 Oct 5. 10.3109/1061186X.2012.729214 23036042

[47] Hillaireau H, Couvreur P (2009) Nanocarriers’ entry into the cell: relevance to drug delivery. Cell Mol Life Sci 66: 2873–2896. 19499185 10.1007/s00018-009-0053-z PMC11115599

[48] Wang J, Sui M, Fan W (2010) Nanoparticles for tumor targeted therapies and their pharmacokinetics. Curr Drug Metab 11: 129–141. 20359289 10.2174/138920010791110827

[49] Haque F, Shu D, Shu Y, Shlyakhtenko LS, Rychahou PG, et al (2012) Ultrastable synergistic tetravalent RNA nanoparticles for targeting to cancers. Nano Today 7: 245–257. 23024702 10.1016/j.nantod.2012.06.010 PMC3458310

[50] Nam HY, Kwon SM, Chung H, Lee SY, Kwon SH, et al (2009) Cellular uptake mechanism and intracellular fate of hydrophobically modified glycol chitosan nanoparticles. J Control Release 135: 259–267. 19331853 10.1016/j.jconrel.2009.01.018

[51] Koo H, Huh MS, Sun IC, Yuk SH, Choi K, et al (2011) In vivo targeted delivery of nanoparticles for theranosis. Acc Chem Res 44: 1018–1028. 21851104 10.1021/ar2000138

[52] Lim SM, Kim TH, Jiang HH, Park CW, Lee S, et al (2011) Improved biological half-life and anti-tumor activity of TNF-related apoptosis-inducing ligand (TRAIL) using PEG-exposed nanoparticles. Biomaterials 32: 3538–3546. 21306770 10.1016/j.biomaterials.2011.01.054

[53] Koppolu B, Bhavsar Z, Wadajkar AS, Nattama S, Rahimi M, et al (2012) Temperature-sensitive polymer-coated magnetic nanoparticles as a potential drug delivery system for targeted therapy of thyroid cancer. J Biomed Nanotechnol 8: 983–990. 23030006 10.1166/jbn.2012.1465

[54] Chen D, Tang Q, Li X, Zhou X, Zang J, et al (2012) Biocompatibility of magnetic Fe(3)O(4) nanoparticles and their cytotoxic effect on MCF-7 cells. Int J Nanomedicine 7: 4973–4982. 23028225 10.2147/IJN.S35140 PMC3446860

[55] Lühmann T, Rimann M, Bittermann AG, Hall H (2008) Cellular uptake and intracellular pathways of PLL-g-PEG-DNA nanoparticles. Bioconjug Chem 19: 1907–1916. 18717536 10.1021/bc800206r

[56] Kozako T, Arima N, Yoshimitsu M, Honda SI, Soeda S (2012) Liposomes and nanotechnology in drug development: focus on oncotargets. Int J Nanomedicine 7: 4943–4951. 23028222 10.2147/IJN.S30726 PMC3446859

[57] Dos Santos T, Varela J, Lynch I, Salvati A, Dawson KA (2011) Effects of transport inhibitors on the cellular uptake of carboxylated polystyrene nanoparticles in different cell lines. PLoS One 6: e24438. 21949717 10.1371/journal.pone.0024438 PMC3176276

