# Supplementary figures and images for: Cellular and Molecular Mechanisms Underlie the Anti-Tumor Activities Exerted by Walterinnesia aegyptia Venom Combined with Silica Nanoparticles against Multiple Myeloma Cancer Cell Types
Source: PLoS One. 2012 Dec 10;7(12):e51661. doi: 10.1371/journal.pone.0051661 (PMC3518476; doi:10.1371/journal.pone.0051661)

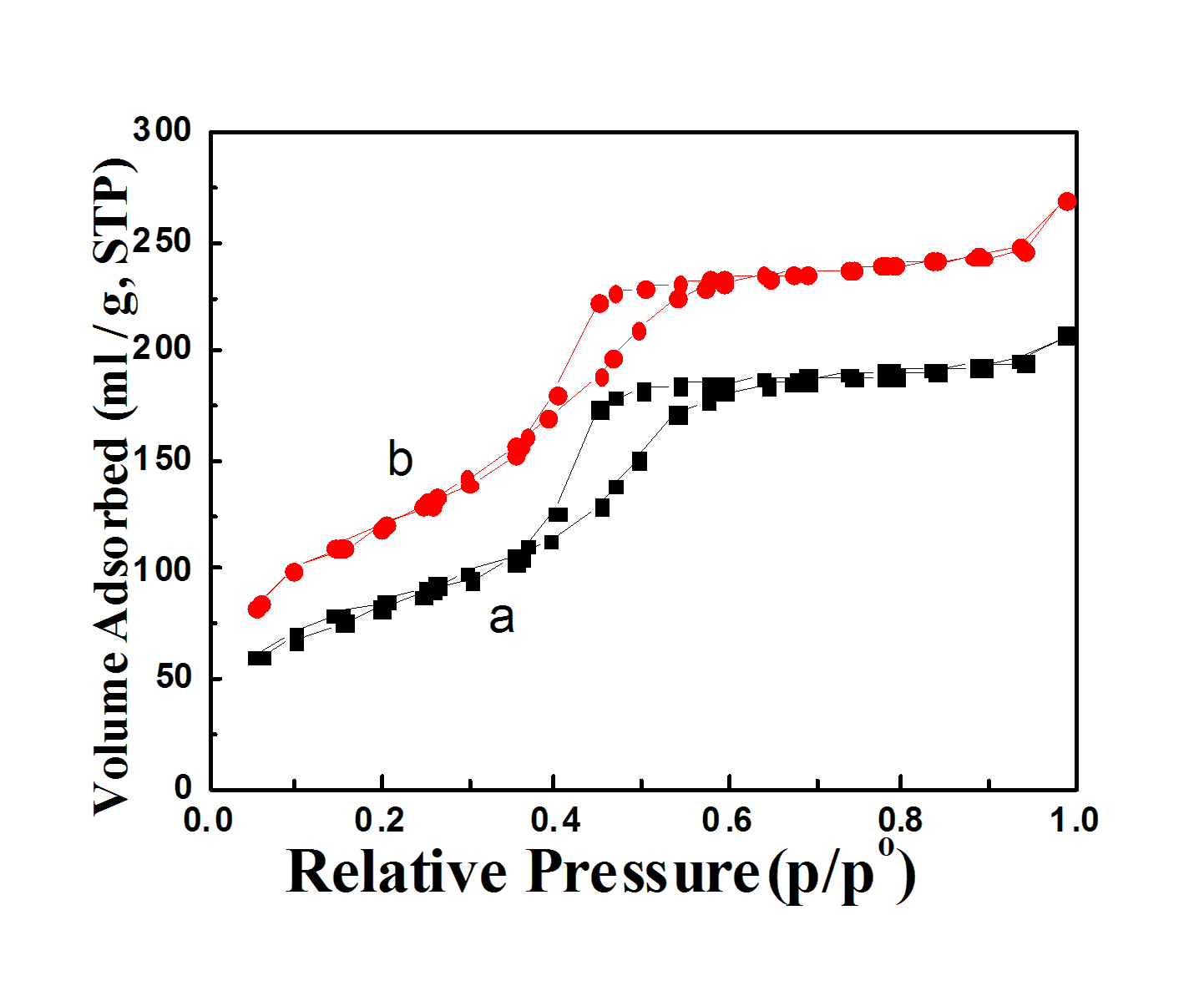

Supplement: Figure S3 — N2 adsorption/desorption isotherms of calcined double mesoporous core-shell silica nanospheres prepared from silica cores formed at different reaction time of (a) 1 h and (b) 6 h. (TIF) [file pone.0051661.s003.tif]

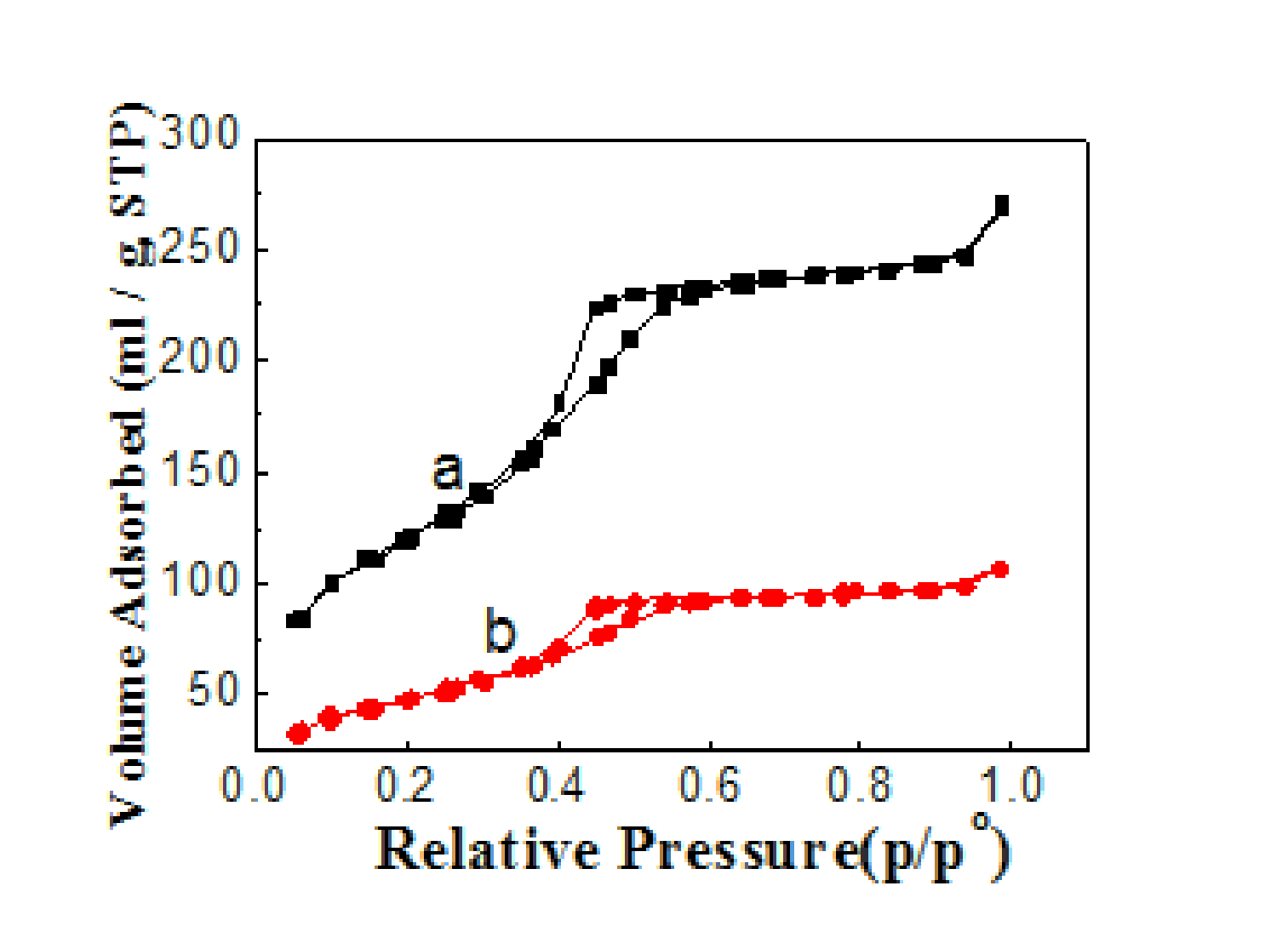

Supplement: Figure S4 — N2 adsorption/desorption isotherms of calcined double mesoporous core-shell silica nanospheres prepared from silica cores formed at 6 h (a) before venom loading and (b) after venom loading. (TIF) [file pone.0051661.s004.tif]
